# Moxa-combustion byproducts improve cognitive function via olfactory-mediated modulation of the GSK-3β/CREB pathway

**DOI:** 10.3389/fnins.2026.1759500

**Published:** 2026-04-01

**Authors:** Jia-Wei Wu, Ya-Jie Liu, Jing He, Yu-Qing Shi, Bai-Xiao Zhao

**Affiliations:** 1 School of Acupuncture-Moxibustion and Tuina, Beijing University of Chinese Medicine, Beijing, China; 2 Xiamen Hospital of Traditional Chinese Medicine, Xiamen, Fujian, China; 3 Langfang Health Vocational College, Langfang, Hebei, China

**Keywords:** Alzheimer’s disease, GSK-3β, moxa-combustion byproducts, olfactory dysfunction, synaptic plasticity

## Abstract

**Objective:**

Olfactory dysfunction in early-stage Alzheimer’s disease (AD) is associated with GSK-3β abnormalities localized in the olfactory bulb. These pathological changes not only compromise hippocampus-dependent learning and memory via synaptic impairments but also contribute to emotional disturbances such as anxiety and depression. Given that moxa-combustion byproducts (MCB), a key therapeutic component of moxibustion, enhance synaptic plasticity and improve cognition, this study aimed to investigate whether MCB ameliorates cognitive deficits by olfactory-mediated modulation of the GSK-3β/CREB pathway.

**Methods:**

Four-month-old APP/PS1 mice received 12-week MCB interventions. Behavioral assessments (Morris water maze, buried food pellet test and open field test) evaluated olfactory and cognitive functions. Pathological changes and synaptic structure in olfactory bulbs and hippocampus were analyzed via hematoxylin–eosin (HE) staining and transmission electron microscopy (TEM). Protein levels of GSK3β, CREB, and c-Fos were quantified by Western blot (WB).

**Results:**

MCB significantly improved spatial learning, memory, and olfactory performance in AD mice. HE staining showed that MCB increased the number of olfactory mitral cells and hippocampal CA1 neurons, and could regulate synaptic plasticity. MCB downregulated GSK-3β while upregulating CREB and c-Fos in both olfactory bulb and hippocampal tissues. The effect of MCB disappeared after olfactory blockade using 3-methylindole, suggesting olfactory-mediated action.

**Conclusion:**

MCB enhances cognitive-olfactory functions and mitigates anxiety- and depression-like behaviors in AD mice, potentially via modulation of GSK-3β/CREB pathway-related proteins in the olfactory system.

## Graphical abstract

**Figure:**
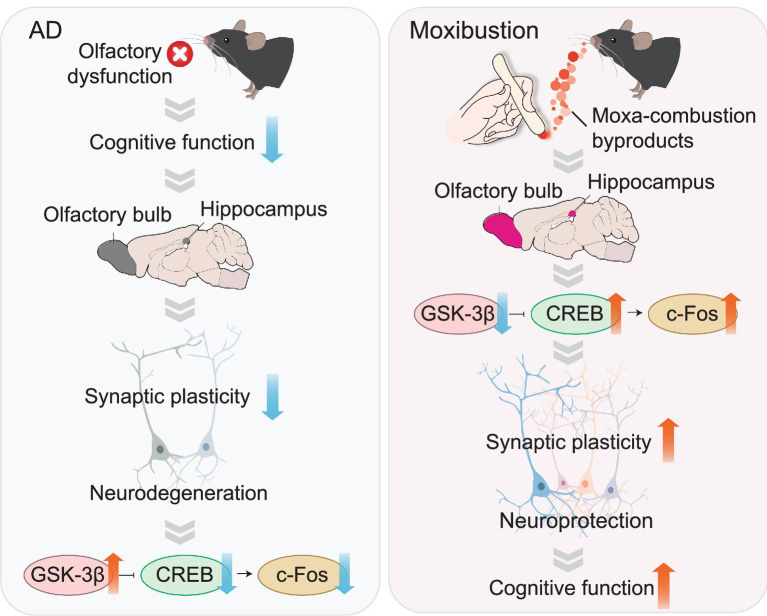
Mechanism diagram of Moxa-combustion byproducts improve cognitive function via olfactory-mediated modulation of the GSK-3β/CREB pathway.

## Introduction

Alzheimer’s disease (AD) is a progressive neurodegenerative disease, its characterized by cognitive decline and impaired learning and memory, as well as neuropsychiatric symptoms such as anxiety and depression. Research demonstrates that olfactory dysfunction manifests in AD patients during initial subjective cognitive decline (Franco et al., 2024; Liao et al., 2024). These alterations precede structural brain changes, memory deficits, and clinical symptom onset. Olfactory and cognitive deficits in AD are closely related to lesions of the olfactory bulb and hippocampus (Attems et al., 2014; Carnemolla et al., 2022; Salimi et al., 2022). As a key connecting the external environment and the brain, the olfactory bulb can directly transmit olfactory stimuli to various regions of the brain (including the hippocampus in the limbic system) through the olfactory pathway. Also, the olfactory pathway has a high degree of anatomical overlap and close neural connectivity with the limbic system, and its pathological damage may exacerbate cognitive decline through hippocampal dysfunction (Kharlamova et al., 2023). Research has shown that olfactory stimuli can promote cognitive function (Liu et al., 2020; Casares et al., 2023). Therefore, improving the cognitive function through olfactory stimulation has become a new idea for preventing AD.

Glycogen synthase kinase-3β (GSK-3β) plays a crucial role in tau phosphorylation, Aβ generation and the progression of neurodegeneration, this disrupts the stability of neuronal microtubules, leading to synaptic dysfunction and ultimately results in neuronal death (Lauretti et al., 2020; Hussain et al., 2025). In addition, GSK-3β is widely distributed in the olfactory bulb, and inhibiting GSK-3β can promote the survival of intermediate neurons in the olfactory bulb and improve olfactory memory (Xu Z. et al., 2013; Hu et al., 2015). It forms the GSK-3β/CREB signaling pathway with the cAMP response element-binding protein (CREB), which regulates learning and memory, synaptic plasticity, and other functions. Therefore, the focus is on the role of GSK-3β in the olfactory bulb and hippocampus, or the key points connecting AD olfaction and memory.

Moxa-combustion byproducts (MCB) can also be termed moxa smoke, have multiple effects such as antioxidant, anti-inflammatory, and neural regulation, been applied in the research of various diseases (Ha et al., 2019; Zhang et al., 2019; Liu et al., 2023), and can ensure the efficacy of moxibustion treatment. Our team’s previous research found that MCB intervention can reduce the deposition of Aβ protein in the olfactory pathway of AD model mice, improve neurotransmitter disorders and synaptic plasticity in the olfactory bulb and hippocampus, enhance olfactory and cognitive functions (Xu H. et al., 2013; Shi et al., 2024; He et al., 2024a,b), suggesting that MCB may exert neuroprotective effects through the olfactory pathway. However, the specific mechanism has not been elucidated.

Therefore, we hypothesize that MCB modulates the expression of GSK-3β/CREB pathway-related proteins through olfactory conduction, which enhances synaptic plasticity and improves spatial memory and olfactory function in AD model mice. This study links the aromatic properties of MCB with the neural repair mechanism driven by GSK-3β/CREB, providing a theoretical basis for odor based intervention in the treatment of neurodegenerative diseases.

## Materials and methods

### Animal

APP/PS1 mice were generated by transgenic techniques introducing the Swedish mutant amyloid precursor protein gene (APP695swe) and the human presenilin 1 gene with exon 9 deletion (PSEN1dE9) into the mouse genome, with a C57BL/6 J genetic background. These mice develop cognitive dysfunction and are widely used in AD research. Four-month-old APP/PS1 mice have been reported to mark the onset of olfactory dysfunction, providing an optimal window for evaluating early intervention (Wu et al., 2013; Li et al., 2019). In this study, four-month-old male APP/PS1 mice were obtained from Beijing Weishang Lituo Technology Co., Ltd. The mice were bred by crossing male and female transgenic founders, followed by genotype identification to confirm the presence of the transgenes. Upon arrival, the mice were randomly divided into the AD model group (AD), rosemary essential oil group (AD + EO), moxa-combustion byproducts group (AD + MCB), olfactory dysfunction + moxa-combustion byproducts group (AD + 3MI + MCB) (*n* = 12 mice/group). Male C57BL/6 mice of the same age and genetic background were used as the blank control group (CG) (*n* = 12 mice). All mice were maintained at 23 ± 2 °C and 60 ± 15% relative humidity with free access to feed and water, controlled environment with a 12-h light/dark schedule. All experimental procedures involving animals were conducted in strict accordance with the National Institutes of Health Guide for the Care and Use of Laboratory Animals and adhered to the ARRIVE guidelines. All animal care and experimental procedures were approved by Animals Care and Use Committee of Beijing University of Traditional Chinese Medicine (Approval No. BUCM-4-2022090501-3084).

### Intervention

**Figure 1:**
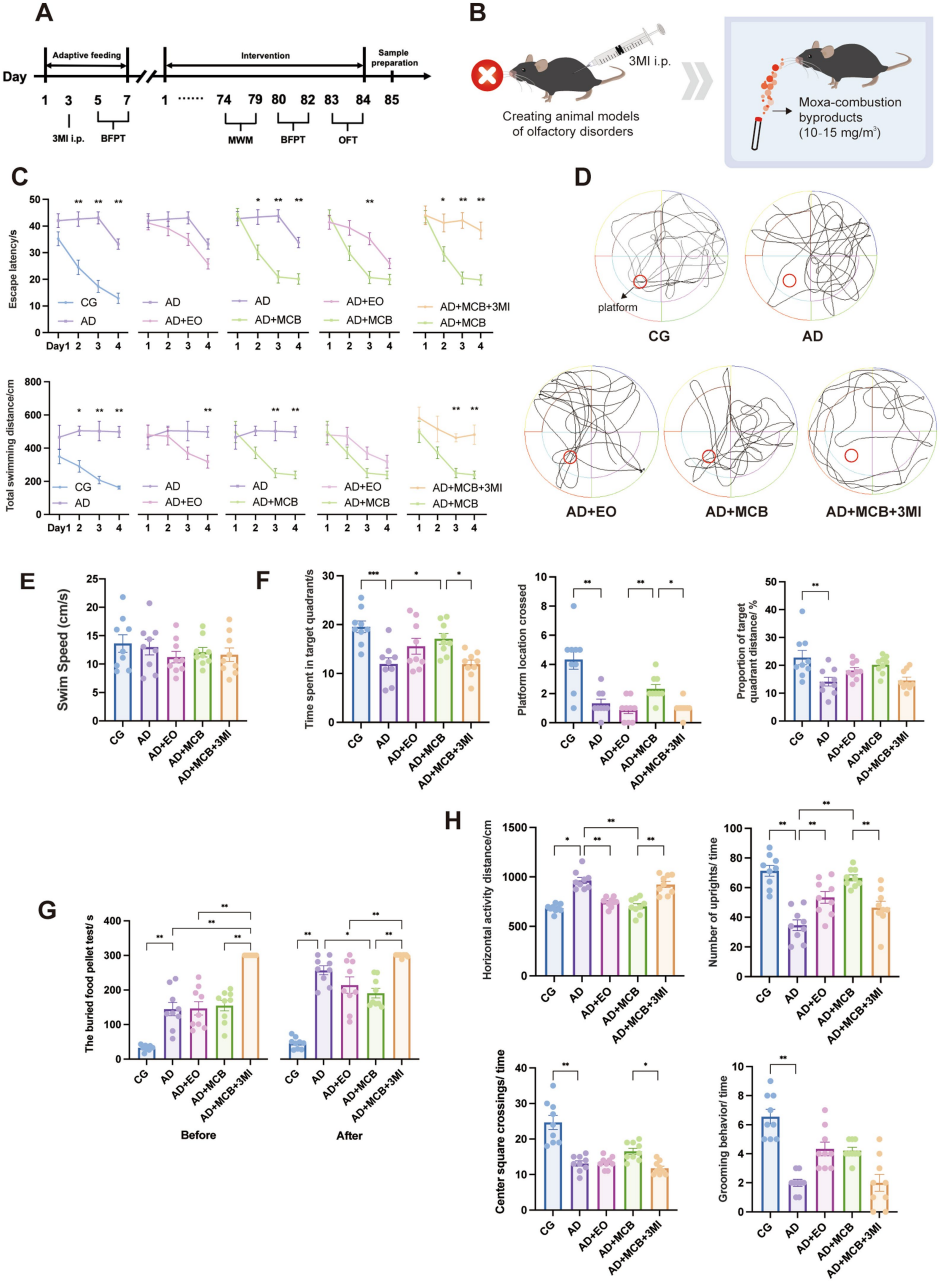
MCB ameliorates cognitive deficits, olfactory dysfunction, and emotional disturbances in APP/PS1 mice. The experimental workflow **(A)** and MCB intervention protocol following olfactory dysfunction modeling **(B)** are presented. Morris water maze results demonstrated progressive reductions in escape latency and swimming distance across training days during the directional navigation test **(C)**, while spatial probe trials revealed representative swimming trajectories **(D)**, consistent mean swimming speeds **(E)**, and quantitative improvements in platform crossings, target quadrant occupancy, and time allocation **(F)**. The food pellet burial test **(G)** demonstrated that MCB reduced food-seeking time. Open field testing **(H)** revealed MCB increased the horizontal activity distance, the number of upright, center square crossings times and grooming behavior, effectively alleviating depressive and anxiety-like behaviors (*n* = 9 mice). AD model group (AD), rosemary essential oil group (AD + EO), Moxa-combustion byproducts group (AD + MCB), olfactory dysfunction + Moxa-combustion byproducts group (AD + 3MI + MCB). Two-way ANOVA with repeated measures was used in **(C)**, Tukey’s HSD was used in **(F–H)**. Data are expressed as mean ± SEM (^*^ *p* < 0.05, ^**^ *p* < 0.01).

The experimental workflow and MCB intervention protocol following olfactory dysfunction modeling are presented in Figures 1A,B. Mice in CG and AD groups were restrained by daily grasping without any intervention. AD + EO group was placed in chamber (50 cm × 40 cm × 30 cm) with 1% rosemary essential oil, and an ultrasonic aromatherapy machine (BENKS, XX01, 50 mL/h) was used for the essential oil inhalation interventions. 1% rosemary essential oil was prepared by adding 0.2 mL of rosemary essential oil (AFU, 2017010366) to 19.8 mL of double-distilled water containing 1% Tween-80 (Coolaber, CT311833100), referring to the literature method (Postu et al., 2020). The generation method of MCB has been improved based on previous literature (Ha et al., 2019). MCB were generated inside the chamber (50 cm × 40 cm × 30 cm) by burning moxa sticks (length, 20–21 cm; diameter, 1.9–2.1 cm; Hubei Li Shizhen Herbal Pieces Co., Ltd., Hubei, China) that are widely used by the Chinese population. The sticks are encased in mugwort floss, which is made from dried mugwort leaves. The concentration of MCB within the chamber was continuously monitored using a light-scattering digital dust meter (DT, Beijing BINTA Green Technology Co., Ltd., Beijing, China). Once the MCB concentration stabilized at 10–15 mg/m^3^, mice in the AD + MCB and AD + 3MI + MCB groups were placed in the chamber for inhalation intervention. All interventions were conducted once daily for 20 min, 6 days per week for 12 weeks. Olfactory dysfunction model preparation method: corn oil (BENCHMARKS, S03GS160014) was used as the solvent, and 3-methylindole (3-MI) (BENCHMARKS, Y18M8C36140) was prepared as a 30 mg/mL solution and injected intraperitoneally into AD + 3MI + MCB group mice at a dose of 300 mg/kg before the start of the intervention.

### Morris water maze

Morris water maze was used to measure the hippocampus-dependent spatial memory. Although mice are nocturnal animals and their active phase occurs during the dark period, all behavioral tests in this study were conducted during the light phase (07:00–19:00) due to practical laboratory scheduling constraints. To minimize potential circadian confounds and better align with the nocturnal nature of mice, the testing room was kept dark during all trials, and all mice were tested at approximately the same time each day with counterbalanced group order. Video analysis system (Chengdu Tame Software Co., Ltd., China) was used to observe and record the swimming pattern of each mouse. The pool was filled with water (21 ± 1 °C). An escape platform (10 cm in diameter) was placed in the pool and the top of the platform was 1 cm below the water surface. Mice were trained for 5 days with 4 trials/day (60 s/trial, 30 min intertrial intervals). If the animal could not find the platform within 60 s, they were placed on the platform for 10 s. On the sixth day, the platform was removed from the pool for the probe test, and the mouse was allowed to swim freely for 1 min.

### The food pellet burying test

The food pellet burying test was conducted in an open-field arena (45 cm × 24 cm × 20 cm) lined with 3 cm of sterile corncob bedding, where a 1 g food pellet was randomly buried 0.5 cm beneath the surface. Prior to testing, mice underwent a 72-h dietary restriction (0.2 g/day with ad libitum water access), and fresh bedding was replaced after each trial to eliminate residual cues. During testing, mice were individually placed into the arena at a standardized starting position (randomized daily) to measure the latency to locate the hidden pellet, defined as the time from placement to forepaw or teeth contact with the pellet, with a maximum cutoff of 300 s. Mice were allowed to consume retrieved pellets, and trials were repeated once daily for three consecutive days at consistent time intervals to ensure temporal uniformity.

### The open field test

The open field test was performed in a semi-enclosed arena (54 cm × 50 cm × 37 cm) constructed with detachable, breathable transparent acrylic panels. The apparatus included: (1) an external infrared sensor array to automatically quantify rearing events (vertical activity); (2) a black metal floor equipped with tactile sensors to record horizontal locomotion distance; and (3) a transparent acrylic cover to minimize environmental interference during testing. A predefined central zone (25 cm × 25 cm) was demarcated within the arena. Prior to testing, mice were acclimated to the environment for 30 min. Each mouse was placed in the lower-left corner of the arena and allowed to freely explore for 5 min during daily trials conducted at consistent time intervals over two consecutive days. Horizontal locomotion and rearing events were automatically recorded by the system, while manual scoring tracked central zone entries and grooming behaviors (e.g., face washing, paw licking, scratching). To eliminate odor cues, the arena was thoroughly cleaned with 75% ethanol and ventilated for 3 min between trials.

### Hematoxylin and eosin staining

Mice were transcardially perfused with cold phosphate-buffered saline (PBS, 10 mM, pH = 7.4), followed by inhalation of 3.5% isoflurane anesthesia. The perfused brain tissues were soaked in 4% paraformaldehyde (Huzhen, HZ100215-250) for at least 24 h, dehydrated in an ascending ethanol series, and equilibrated with xylene (BioDee, 534056-20L). Then, tissues were embedded in paraffin and cut into 5 μm coronal sections through the olfactory bulb and hippocampus. For the olfactory bulb, sections containing the mitral cell layer were selected, corresponding approximately to Bregma +4.28 mm to +3.56 mm; for the hippocampus, sections corresponding to Bregma −1.70 mm to −2.30 mm, including the CA1 region. Three mice per group were analyzed, and three randomly selected fields per region were observed. After conventional dewaxing and hydration, sections were stained with hematoxylin and eosin (Baiaolaibo, L10532, SNM352). The pathomorphological changes were observed by microscope using a double-blind method.

### Transmission electron microscope

Following transcardial perfusion with 4% paraformaldehyde, brain tissues were fixed with 2% (w/v) glutaraldehyde (Sigma-Aldrich, HZ131278-250) in 0.1 M phosphate buffer (50 mM Na2HPO4, Sigma-Aldrich 71,649; 50 mM NaH2PO4, Sigma-Aldrich 71,507; pH 7.4) for 1 h at room temperature and stored overnight at 4 °C. Samples were subsequently rinsed three times with 0.1 M phosphate buffer and post-fixed in 1% (w/v) osmium tetroxide (Ted Pella, 18,459) containing 0.8% (w/v) potassium ferrocyanide (Sigma-Aldrich, P3289) for 2 h at 4 °C. After three washes with distilled water, tissues were dehydrated through a graded ethanol series (50, 70, 90, 100%) and embedded in EMbed 812 resin (Electron Microscopy Sciences, 13,940). Ultrathin sections (70 nm) were cut using a Leica UC7 ultramicrotome, stained with uranyl acetate and lead citrate, and examined under a JEOL JEM-1400 transmission electron microscope operated at 80 kV.

### Western blot

Olfactory blub and hippocampal tissues (20 mg) were lysed in RIPA lysis buffer (Coolaber, SL1020). BCA protein assay kit determine the protein concentration. Equal amount of protein was loaded onto 8–15% SDS-PAGE gal, and transferred to PVDF membranes. After blocking in 5% skim milk in TBS-T buffer at room temperature for 1 h, the membranes were followed by incubation with primary antibody at 4°C overnight: anti-GSK-3β (1:2000, Abcam, United States); anti-CREB (1:2000, Abcam, United States); anti-c-Fos (1:1000, Abcam, United States); anti-GAPDH (1:2000, Abcam, United States). After being washed in TBS-T buffer, the membranes were incubated with horseradish peroxidase (HRP) by anti-rabbit HRP (Amersham Pharmacia, United States) at room temperature for 1 h. Then, the blots were developed by Immobilon Western ECL solution (Merck Millipore, United States) and immunoreactive bands were visualized using an Image Station 4000MM (#745,280; Kodak, Japan).

### Statistics

Statistical analysis was performed with software the Statistical Package for the Social Sciences version 25.0 (SPSS, Inc., Chicago, IL, United States), and data were expressed as mean ± standard error ( $\bar{x}$ ± SEM). We applied predetermined exclusion criteria to ensure data quality. Three mice per group were excluded from the behavioral analysis due to excessive floating behavior (immobility > 50% of trial time) or technical issues during video tracking (e.g., tracking loss). For escape latency and swimming distance in the MWM test, two-way repeated-measures ANOVA. One-way ANOVA was used after the test of normal distribution and homogeneity of variance, and Tukey’s HSD post-hoc test was used for pairwise comparisons. If there was a non-normal distribution or heterogeneity of variance for the data, a Kruskal-Wallis test would be used. Statistical significance was set to *p* < 0.05, while highly statistical significance was set to *p* < 0.01.

## Results

### MCB ameliorates cognitive deficits, olfactory dysfunction, and emotional disturbances in APP/PS1 mice

In order to evaluate the therapeutic effect of MCB on improving spatial learning and memory, the Morris water maze was used to detect the learning and memory functions of APP/PS1 mice (Figures 1C–F). After 3 months of intervention with MCB and rosemary essential oil, the mice were tested. In the 4-day directional navigation experiment, the latency and path length (Figure 1C) to reach the platform were reduced. Notably, MCB-treated mice exhibited significantly shorter latencies compared to AD mice on day 2 (*p* = 0.015), day 3 (*p* = 0.001), and day 4 (*p* = 0.001). For total swimming distance, MCB treatment significantly reduced path length compared to AD mice on day 3 (*p* = 0.001), and day 4 (*p* = 0.001). While the path length of EO group mice were significantly reduced on day 4 (*p* = 0.001) compared to AD mice. The space exploration experiment revealed substantial spatial memory deficits in AD group relative to wild-type C57BL/6 mice, as quantified by reduced proportion of target quadrant distance (*p* = 0.0038), time spent in target quadrant (*p* = 0.0007), and platform location crossed times (*p* = 0.0177), while there was no difference in the swimming speed of mice in each group (*F* = 0.65, *p* = 0.6301), excluding the influence of the difference in exercise ability on the experimental results. Strikingly, MCB treatment increased time spent in target quadrant (*p* = 0.0383) compared to AD group, while MCB-treated mice demonstrated superior performance in platform location crossed times to EO-treated APP/PS1 mice (*p* = 0.0065). These findings collectively demonstrate that MCB effectively rescue memory retention impairments in APP/PS1 mice.

In order to verify the effect of MCB on olfaction, we evaluated the olfactory function by buried food pellet test (Figure 1G). We first compared the groups before intervention (*F* = 45.7, *η^2^p* = 0.82), the AD mice exhibited significantly longer latencies to locate the buried food pellet compared to the CG group (*p* = 0.0001). The AD+3MI + MCB group showed prolonged latency versus the model group (*p* = 0.0001), while no significant differences were observed between the EO/ MCB groups and the AD group (*p* > 0.05). After 3 months of intervention, there were significant differences among the groups (*F* = 49.2, *η^2^p* = 0.831). AD groups maintained significantly longer latencies than the control group (*p* = 0.0001). Notably, the MCB group demonstrated a significant reduction in latency compared to AD group (*p* = 0.0125). After olfactory dysfunction by 3-MI, the mice exhibited markedly longer latency than MCB group (*p* = 0.0001), while no difference was detected between the EO and MCB groups (*p* = 0.7442). This shows that MCB can reduce the time of burned food pellet test and improve olfactory function.

AD often causes adverse emotional reactions such as anxiety and depression after olfactory dysfunction. Then we used the open field test to evaluate the effect of MCB on the emotional state and autonomic motor ability of mice (Figure 1H). Compared with CG group, the horizontal activity distance of AD group mice was significantly increased (*p* = 0.0001), and the number of upright (*p* = 0.0001), center square crossings times (*p* = 0.0001) and grooming behavior (*p* = 0.0001) were significantly reduced. Compared with AD group, the EO group (*p* = 0.0001) and the MCB group (*p* = 0.0001) showed a significant decrease in horizontal activity distance, a significant increase in the number of upright (AD vs. EO: *p* = 0.0053; AD vs. MCB: *p* = 0.0001). Compared with the MCB group, the AD+3MI + MCB group showed a significant increase in horizontal activity distance (*p* = 0.0001), and a significant decrease in upright (*p* = 0.0027), center square crossings times (*p* = 0.0259). The above results suggest that MCB intervention has specific advantages in regulating emotional behavior and enhancing exploratory ability.

### MCB preserves neuronal morphology in hippocampus and olfactory bulb

**Figure 2:**
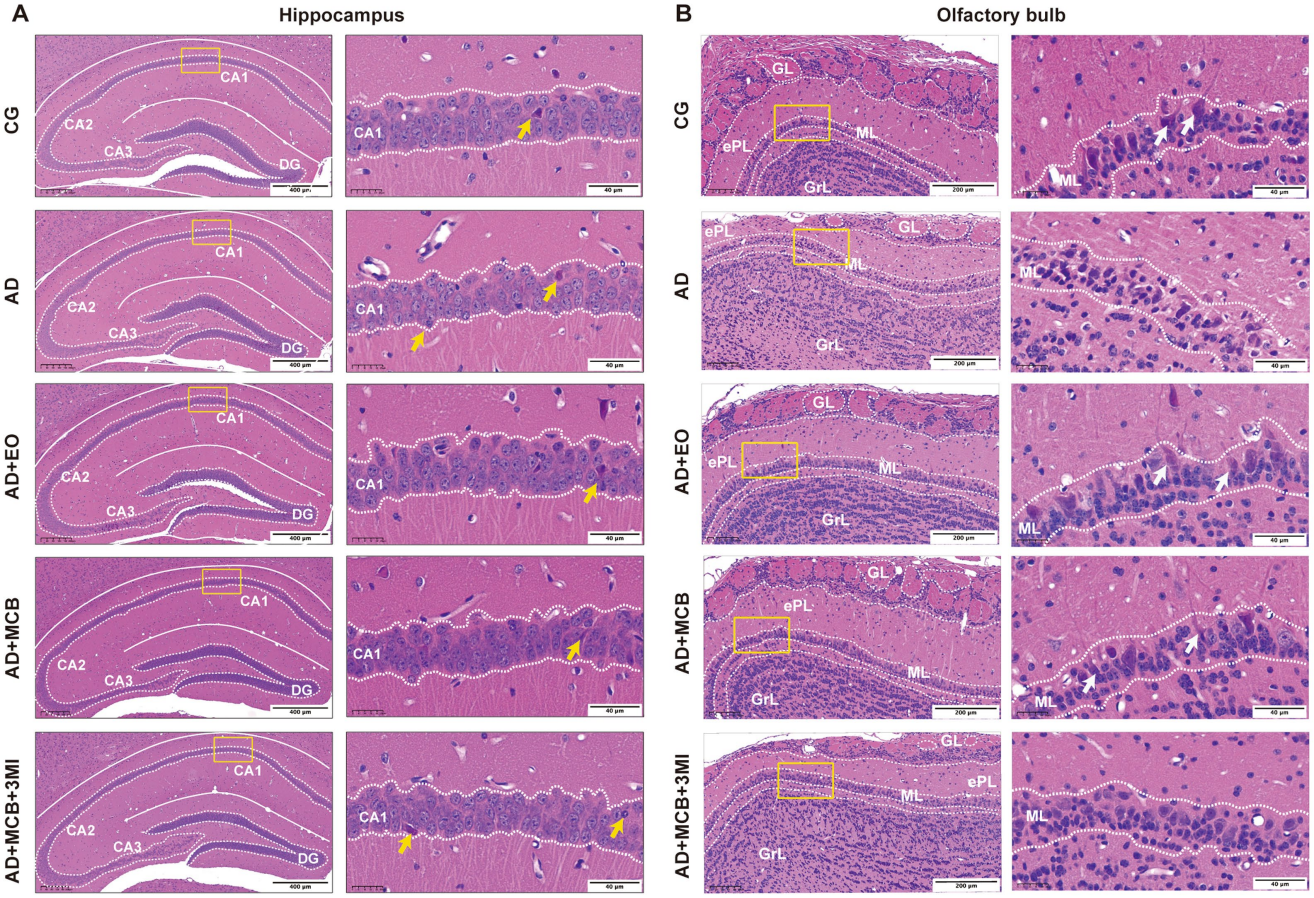
MCB preserves neuronal morphology in the hippocampus and olfactory bulb of APP/PS1 mice. Hippocampal CA1 and olfactory bulb histopathological analysis via hematoxylin–eosin (HE) staining. **(A)** Hippocampal CA1 identified pyknotic nuclei (yellow arrows), reflecting neurodegenerative changes (*n* = 3 mice). **(B)** Olfactory bulb indicated that MCB ameliorated pathological alterations in the olfactory bulb and increased mitral cell counts, white arrows indicate mitral cells, GL, glomerular layers; ePL, external plexiform layers; ML, mitral layers; GrL, granule layers, magnification: (left) × 200, Scale bar = 400 μm; (right) × 800, Scale bar = 40 μm (*n* = 3 mice). AD model group (AD), rosemary essential oil group (AD + EO), Moxa-combustion byproducts group (AD + MCB), olfactory dysfunction + Moxa-combustion byproducts group (AD + 3MI + MCB).

HE staining revealed distinct hippocampal CA1 neuronal alterations across experimental groups (Figure 2A). wild-type C57BL/6 mice neurons displayed tightly packed, orderly arrangements with intact laminar organization, uniform cytoplasmic staining, and clearly visible nuclei devoid of pyknosis. In contrast, the AD group exhibited disorganized architecture, sparse neuronal distribution, reduced laminar layers, cytoplasmic hyperchromasia, interstitial edema, and frequent nuclear pyknosis. EO group showed partial structural disruption, moderate laminar thinning, uneven cytoplasmic staining with focal pallor, and rare pyknotic nuclei, while the MCB group demonstrated relatively preserved neuronal alignment, improved laminar organization, homogeneous cytoplasmic staining, and infrequent nuclear pyknosis, despite persistent mild edema and sporadic degeneration. These demonstrated MCB could protect the neuron in the CA1 region of hippocampus at some extent.

The olfactory bulb is the first relay station in the olfactory system to integrate and transmit olfactory information, so we observed the morphological changes of the olfactory bulb by HE staining in APP/PS1 mice (Figure 2B). Compared with the CG group, the model group exhibited loose and disorganized cellular arrangement, indistinct lamellar boundaries with reduced thickness, shrinkage of the olfactory nerve layer and olfactory bulb volume, and decreased distribution density of peribulbar cells and mitral cells. In contrast, the MCB group displayed tightly arranged cellular layers, a thickened yet loosely distributed olfactory nerve layer, slight atrophy of synaptic glomerular cells, and densely distributed peribulbar cells and mitral cells in increased numbers. The EO group demonstrated relatively intact layered cellular organization with mild atrophy of synaptic glomerular cells. Conversely, the AD+3MI + MCB group showed loose cellular arrangement, atrophy of both olfactory nerve layer and synaptic glomerular layer cells, and a marked reduction in peribulbar cell population. This indicates that MCB can improve the morphology and structure of olfactory bulb.

### MCB alters synaptic plasticity and modulates the GSK-3β/CREB pathway

**Figure 3:**
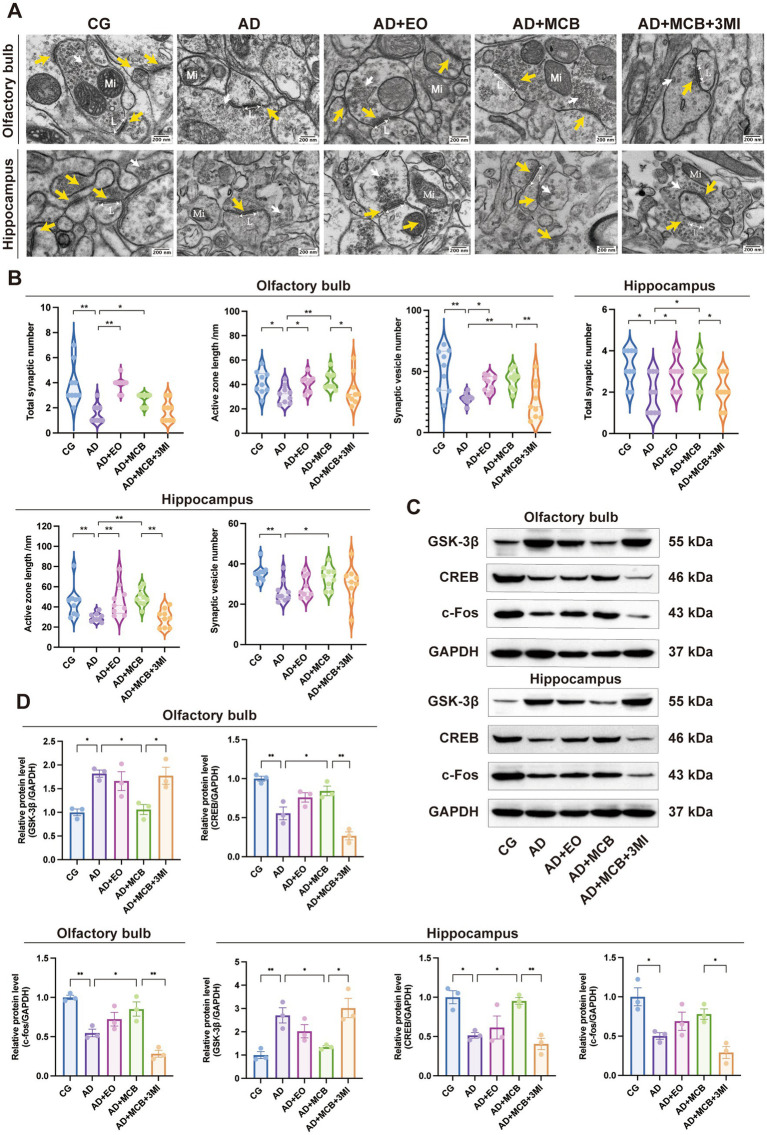
MCB alters synaptic plasticity and activates the GSK-3β/CREB pathway. Transmission electron microscopy **(A,B)** showed that MCB could increase the number of synapses, the length of active regions and the number of vesicles in the olfactory bulb and hippocampus. The yellow arrows indicated the synaptic structure, the white arrows indicated the synaptic vesicles, L, the length of active regions; Mi, mitochondria (*n* = 3 mice). Western blot **(C,D)** showed that MCB could reduce the protein expression of GSK-3β and increase the protein expressions of CREB and c-Fos (*n* = 6 mice). AD model group (AD), rosemary essential oil group (AD + EO), Moxa-combustion byproducts group (AD + MCB), olfactory dysfunction + Moxa-combustion byproducts group (AD + 3MI + MCB). Kruskal–Wallis test was used in **(B)**. Tukey’s HSD was used in **(D)**. Data are expressed as mean ± SEM (^*^ *p* < 0.05, ^**^ *p* < 0.01).

Synapse is the key part of information transmission, so we observed the changes of synaptic structure in olfactory bulb and hippocampus of APP/PS1 mice by transmission electron microscope (Figures 3A,B). In the olfactory bulb, the CG group exhibited intact synaptic structures with abundant organelles. Compared to CG group, the AD group showed significant reductions in synaptic number (*p* = 0.001), active zone length (*p* = 0.024), and synaptic vesicle number (*p* = 0.001). Conversely, both the EO and MCB groups displayed marked increases in these parameters relative to AD group (synaptic number (EO: *p* = 0.001; MCB: *p* = 0.042), active zone length (EO: *p* = 0.034; MCB: *p* = 0.008), and synaptic vesicle number (EO: *p* = 0.018; MCB: *p* = 0.004)). Notably, the AD+3MI + MCB group exhibited reduced active zone length (*p* = 0.034) and synaptic vesicle number (*p* = 0.008) relative to the MCB group. In the hippocampus, CG group demonstrated higher synaptic density with well-defined structures. Synaptic number (*p* = 0.004), active zone length (*p* = 0.005), and synaptic vesicle number (*p* = 0.002) were significantly decreased in AD group versus CG group. While both the EO and MCB groups displayed marked increases in these parameters relative to AD group (synaptic number (EO: *p* = 0.037; MCB: *p* = 0.017), active zone length (EO: *p* = 0.003; MCB: *p* = 0.001), and synaptic vesicle number (MCB: *p* = 0.016)). And the AD+3MI + MCB group exhibited reduced synaptic density (*p* = 0.043) and active zone length (*p* = 0.001) relative to the MCB group.

Then we analyzed the expression of GSK-3β/CREB signaling pathway related proteins in olfactory bulb and hippocampus by Western blot (Figures 3C,D). In the olfactory bulb, compared to CG group, the AD group exhibited significantly upregulated GSK-3β (*p* = 0.0119) and downregulated CREB (*p* = 0.0025) and c-Fos (*p* = 0.0040) protein levels, whereas MCB group showed significant reductions in GSK-3β protein expression (*p* = 0.0188) and increases in CREB (*p* = 0.0414) and c-Fos protein levels (*p* = 0.0467) compared to AD group. Though the AD+3MI + MCB group displayed reversed trends with increased GSK-3β (*p* = 0.0272) and decreased CREB (*p* = 0.0003) and c-Fos (*p* = 0.0007) compared to MCB group. Similarly, in the hippocampus, AD group showed elevated GSK-3β (*p* = 0.0099) and reduced CREB (*p* = 0.0164) and c-Fos (*p* = 0.0162) versus CG group, while MCB group attenuated GSK-3β (*p* = 0.0377) and enhanced CREB (*p* = 0.0300) expression compared to AD group, but the AD+3MI + MCB group exhibited increased GSK-3β (*p* = 0.0109) and decreased CREB (*p* = 0.0076) and c-Fos (*p* = 0.0183) relative to MCB group. Those results suggest that intervention of MCB modulates the expression of GSK-3β/CREB pathway-related proteins.

## Discussion

In the early stage of AD, neurodegenerative changes have occurred in the olfactory pathway. The lesions extensively involve the olfactory bulb and olfactory cortex. Moreover, olfactory dysfunction, such as impaired odor threshold, odor recognition, and odor cognitive memory, are early symptoms of AD patients (Mantovani et al., 2024). It is positively correlated with cognitive impairment and has been classified as one of the early clinical diagnostic indicators of AD (Fukumoto et al., 2022). It is suggested that intervention before early olfactory lesions may help prevent or delay the progression of AD. Specific odor stimulation improves learning memory by ameliorating abnormal neuronal activity and repairing synaptic plasticity through the olfactory pathway, this provides a potential intervention strategy for AD treatment. In this study, we found that MCB as an odor stimulus significantly improved olfactory deficits and spatial memory deficits in AD model mice, while effectively maintaining the morphological integrity and synaptic function of neurons in the olfactory bulb and hippocampal CA1 region. The mechanism of action may involve promotion of GSK-3β/CREB signaling pathway. In addition, 3-MI olfactory blockade eliminated the neuroprotective effect of MCB, further confirming that the olfactory pathway is a key pathway of action for their function.

Cognitive impairment and olfactory dysfunction in AD are intimately linked to reduced neuron numbers and lower synaptic plasticity (Fu et al., 2017). In AD, neurofibrillary tangles (NFTs) and senile plaques (SPs) involve brain areas selectively and expand throughout disease progression. For instance, NFTs first form in the entorhinal cortex (EC) during early AD (Braak stages I–II). By stages III–IV, they spread widely in the EC and hippocampal CA1 region (Braak and Braak, 1991). However, in some AD patients, the olfactory bulb has been damaged at Braak stage 0 or I, and autopsy results revealed that AD patients have degenerative lesions in their olfactory bulbs. NFTs appear in the olfactory bulbs and tracts at a time similar to that in the EC. This indicates that the olfactory bulb may be one of the first brain regions involved in the process of AD degeneration (Christen-Zaech et al., 2003). Although the research results about the pathological changes of the olfactory bulb, the first level center of the olfactory system, in the occurrence and development of AD are not consistent, it is clear that the brain regions that are most severely affected are those linked to the olfactory system. Moreover, before AD pathology becomes detectable, functional and structural brain abnormalities already occur. MRI studies identified issues in the olfactory pathway, including reduced axonal transport efficiency, abnormal projections and connections of olfactory neurons, synaptic protein loss, and disrupted neural networks (Mucke et al., 2000; Li et al., 2009; Kim et al., 2011; Cao et al., 2012). These disruptions involved suppressed long-term potentiation (LTP), increased long-term depression (LTD), decreased dendritic spine density, altered synaptic transmission and structural changes in the olfactory bulb (Sengoku et al., 2008; Shankar et al., 2008). This aligns with our experiment’s observed olfactory dysfunction and synaptic damage in the olfactory bulb and hippocampus.

GSK-3β is widely recognized as a critical kinase involved in the hyperphosphorylation of tau protein (Rekha et al., 2025). Studies indicate that GSK-3, which comprises two subtypes (GSK-3α and GSK-3β), is upregulated in the central nervous system, with GSK-3β playing a central role in AD pathogenesis (Sayas and Ávila, 2021). and overexpressed GSK-3β is closely associated with AD and olfactory impairment (Kumari et al., 2023; Cheng et al., 2024). Elevated GSK-3β expression has been shown to promote tau hyperphosphorylation at multiple AD-related epitopes, contributing to microtubule destabilization and NFTs formation which in turn leads to synaptic loss (Li et al., 2023; Sayas and Ávila, 2021). Some researchers hypothesize that GSK-3β dysfunction precedes the onset of AD pathology and subsequently engages in a vicious cycle with disease progression after pathological features emerge (Lauretti et al., 2020). In this experiment, MCB intervention was able to downregulate GSK-3β protein expression in the olfactory bulb and hippocampal tissues of the AD model mice, which in line with the findings of Hu et al. (2015). Given the established role of GSK-3β in synaptic integrity, the reduction of GSK-3β expression by MCB may contribute to the preservation of synaptic ultrastructure observed in this study, as evidenced by increased synaptic number, elongated active zone length, and enhanced synaptic vesicle number in the transmission electron microscopy analysis.

Research has demonstrated that GSK-3β serves as an essential kinase regulating the transcription of CREB (Wu et al., 2018). We hypothesize that the early-onset olfactory and learning-memory impairments in AD are associated with neuronal loss and dysregulation of the GSK-3β/CREB signaling pathway. Synaptic loss and plasticity, along with memory, are also linked to the CREB signaling pathway. *In vivo* studies have demonstrated that upregulating CREB ameliorates age-related cognitive decline. Furthermore, CREB-dependent transcription of genes c-Fos (Miyashita et al., 2018). Combined with the results of this experiment, CREB protein expression was reduced in the olfactory bulb and hippocampal tissues of APP/PS1 mice, which was resonated with the experimental results of Yuan et al. (2021). Following MCB intervention, CREB protein expression was significantly upregulated, indicating that MCB partially modulates the expression of GSK-3β/CREB pathway-related proteins, which aligns with the HE staining and electron microscopy observations in this study. Jaworski et al. (2018) have emphasized that the activity of c-Fos-activated transcription factors is critically involved in long-term synaptic plasticity, learning, and memory. This mechanistic finding aligns with the study by Peng et al. (2017), who reported that suppressing GSK3β activity and enhancing c-Fos expression mitigated hippocampal pathological progression and improved spatial memory deficits in AD animal models. However, our study links the olfactory stimulation of MCB with the modulation of pathway-related proteins, providing a new target for non-pharmacological intervention in AD.

The transmission of olfactory signals occurs through synaptic connections. When odorant molecules bind to olfactory receptors in the nasal mucosa, electrical signals are transmitted from neuronal axons to the glomerular layers (GL) of the olfactory bulb. These layers serve as the site where dendrites of mitral/tufted cells form synaptic connections (Egger and Kuner, 2021). Mitral cells, the primary output neurons of the olfactory system, project olfactory information to the olfactory cortex (e.g., anterior olfactory nucleus, piriform cortex, entorhinal cortex, hippocampus, etc.) (Wang et al., 2024). Currently, therapeutic drugs targeting the olfactory circuit for AD remain scarce, with essential oil aromatherapy being the most frequently reported intervention in existing studies. Research shows sniffing rosemary essential oil has cognitive-enhancing effects, and it can relieve anxiety, depression and other emotions. Therefore, it was selected as a positive control drug for reference in this paper (Moss and Oliver, 2012; Pengelly et al., 2012). In this study, the open field experiment of MCP group crosses the central area more times than that of essential oil group. This may be related to the synergistic effect of the complex components of MCB, including 1,8-cineole, camphor, thujone, β-caryophyllene, and caryophyllene oxide, which fall into categories like alcohols (phenols), terpenoids and their derivatives, and alkenes. These substances have a variety of biological activities, for example, 1,8-cineole can reduce oxidative stress and improve cognitive function (Bahrami et al., 2023); β-caryophyllene has been shown to have anti-inflammatory and neuroprotective pharmacological activities (Machado et al., 2018). Studies have shown that rosemary essential oil can improve cognitive function by increasing the content of 1,8-cineole in serum and increasing the level of deoxyhemoglobin in cerebral blood vessels (Moss and Oliver, 2012; Moss et al., 2018). In contrast, the therapeutic advantage of MCB may stem from its multi-component synergy. However, the bioactive molecules that play a specific therapeutic role in MCB still need further screening. 3-MI generates toxic products directly damage olfactory neurons and supporting cells while reducing olfactory input, it is a commonly used drug to induce olfactory impairment (Kim et al., 2010). In this study, after 3-MI blocked olfaction in mice, the neuroprotective effect of MCB completely disappeared, which is consistent with the view that loss of olfactory input exacerbates AD pathology (Marin et al., 2018). In addition, it also verified that the action of MCB may be through the olfactory pathway.

However, recent analytical studies have identified potentially hazardous substances in MCB, which has raised growing concerns regarding their biosafety profiles (Xu et al., 2020). It has been reported that moxibustion may exert significant effects on certain patients with chronic pharyngitis, inducing coughing due to hypersensitivity to MCB, yet these symptoms gradually ameliorate with improved air circulation (Park et al., 2010). Although some studies have confirmed the safety of MCB in animal models (no lung function injury was observed) (He et al., 2019), it should be noted that the MCB concentration in the experiment (10 ~ 15 mg/m^3^) was significantly higher than the clinical exposure level (about 3.54 mg/m^3^). This higher concentration in this study was chosen to maximize the detection of therapeutic effects within a short experimental period, given the metabolic differences between rodents and humans. But the potential risk of long-term high concentration exposure still needs to be vigilant. In addition, the complex components of MCB may contain controversial substances (such as polycyclic aromatic hydrocarbons), and its clinical applicability needs to be clarified in combination with toxicological analysis in the future.

Notably, as core bioactive constituents of moxibustion therapy, MCB may delay AD pathological progression through olfaction-mediated molecular regulation, particularly via GSK-3β suppression and CREB/c-Fos pathway-related proteins modulation, providing a theoretical basis for odor-based interventions in neurodegenerative diseases. That is a very novel point of view. However, this study also has some limitations. The expression of Aβ or tau protein was not detected in this study, because this article focuses more on the olfactory pathological changes before the emergence of AD pathology. Furthermore, the current work specifically addresses olfactory-hippocampal interactions, future studies will expand to olfactory pathway-associated brain regions (e.g., anterior olfactory nucleus, piriform cortex, and entorhinal cortex) to comprehensively elucidate neural circuit mechanisms. Additionally, the lack of direct compositional analysis of MCB in this study is a limitation. Further experiments are needed to identify and validate the specific bioactive compounds responsible for the observed effects. Finally, while the use of only male mice ensured consistency with our previous studies and minimized hormonal variability, it limits the generalizability of our findings; therefore, future studies will include both sexes to explore potential sex-dependent effects of MCB intervention.

## Conclusion

This study demonstrates that MCB improve cognitive through olfactory activation. Notably, it is links MCB’s aromatic properties to the GSK-3β/CREB mechanism, advancing odor-based therapeutic strategies for neurodegenerative diseases. Despite the compositional complexity and safety controversy, the potential of MCB to improve AD pathology through olfactory pathways deserves further exploration and may open up new avenues for non-pharmacological intervention in AD.

## Data Availability

The original contributions presented in the study are included in the article/supplementary material, further inquiries can be directed to the corresponding author.
